# Interictal thermal pain hypersensitivity and central sensitization in migraine patients: a case–control study using quantitative sensory testing

**DOI:** 10.1186/s13005-026-00614-0

**Published:** 2026-03-20

**Authors:** Keisuke Suzuki, Shiho Suzuki, Yasuo Haruyama, Mukuto Shioda, Saro Kobayashi, Ryotaro Hida, Koichi Hirata

**Affiliations:** 1 Department of Neurology, Dokkyo Medical University, 880 Kitakobayashi, Shimotsuga, Mibu, Tochigi 321-0293 Japan; 2 Department of Public Health, Dokkyo Medical University School of Medicine, Mibu, Japan

**Keywords:** Migraine, Central sensitization, Quantitative sensory testing

## Abstract

**Background:**

Altered pain processing and central sensitization are key mechanisms underlying migraine. Although quantitative sensory testing (QST) objectively assesses sensory thresholds, its clinical implications in migraine patients are unclear. We hypothesized that migraine patients have lower thermal pain thresholds than controls, reflecting sensory hypersensitivity associated with central sensitization–related features.

**Methods:**

We conducted a single-center case–control study including 130 patients with migraine and 130 sex-matched healthy controls. Thermal QST was performed during headache-free interictal periods using Intercross-220, assessing the heat detection threshold (HDT), cold detection threshold (CDT), thermal sensory limen (TSL), heat pain threshold (HPT), and cold pain threshold (CPT). Symptoms associated with central sensitization were assessed using the Japanese version of the Central Sensitization Inventory (CSI), a self-report questionnaire.

**Results:**

Compared with healthy controls, migraine patients had a significantly lower HPT and higher CPT, indicating hypersensitivity to thermal pain, as well as altered HDTs and CDTs, while the TSL did not differ between groups. The CSI score was significantly correlated with migraine-related disability, interictal burden, ictal allodynia, sleep disturbance, and depressive symptoms. CSI scores were also significantly associated with QST pain-related parameters, whereas other clinical measures were not significantly correlated with sensory thresholds. QST-defined interictal allodynia was more prevalent in migraine patients than in controls (19.2% vs. 8.5%). A CSI score ≥ 40, indicating a high burden of central sensitivity–related symptoms, was observed in 30.8% of migraine patients and 0.8% of healthy controls. In migraine patients, a CSI score ≥ 40 identified QST-defined allodynia with 100% sensitivity and 85.7% specificity.

****Conclusions**:**

Patients with migraine exhibit interictal hypersensitivity to thermal pain. The CSI may serve as a useful patient-reported tool for identifying symptom burden associated with central sensitization-related sensory hypersensitivity in migraine patients.

**Supplementary Information:**

The online version contains supplementary material available at 10.1186/s13005-026-00614-0.

## Introduction

Migraine is a neurological disorder affecting more than one billion people worldwide, with an annual prevalence of approximately 15%, peaking among individuals in their 30s to 40s. Migraine attacks last from 4 to 72 h and are accompanied by symptoms such as photophobia, phonophobia, and nausea. Because the pain is aggravated by physical activity and patients often experience anticipatory anxiety even between attacks, migraine causes a significant impairment in daily functioning [1]. The trigeminovascular theory is supported by the pathogenesis of migraine, suggesting that neuropeptides released from trigeminal nerve endings in response to as yet unknown triggering factors induce neurogenic inflammation characterized by vasodilation and plasma protein extravasation [2].

The neuropeptides released from trigeminal nerve endings include calcitonin gene-related peptide (CGRP), whose concentrations increase in both blood and saliva during migraine attacks. In addition to peripheral sensitization of the trigeminovascular system, central sensitization involving the thalamus, hypothalamus, and sensory cortex is thought to contribute to the chronification and exacerbation of migraine [3]. Central sensitization refers to an abnormal state of pain transmission from the body through the spinal cord, brainstem, thalamus, and cerebral cortex (particularly the primary somatosensory cortex), in which mild stimuli are perceived as painful and pain responses are amplified. Cutaneous allodynia is an abnormal perception in which normally nonpainful stimuli are perceived as painful, and it represents a representative manifestation of central sensitization in migraine patients [4]. In migraine patients, central sensitization has been implicated in the development of cutaneous allodynia and disease chronification [5–7].

The Central Sensitization Inventory (CSI) [8] is a commonly used assessment tool for symptoms associated with central sensitization syndromes, whereas the Pain Severity Questionnaire [9] evaluates pain severity. However, studies evaluating the relationship between actual sensory thresholds and central sensitization are not readily available. Following the administration of galcanezumab, a CGRP monoclonal antibody, responders had significantly higher baseline thermal pain thresholds than nonresponders did, as assessed by quantitative sensory testing (QST) [10]. We hypothesized that compared with healthy individuals, migraine patients have lower pain thresholds for heat and cold sensations and that this reduction in pain threshold is correlated with central sensitization-related symptoms. This study investigated the associations between thermal QST thresholds, headache-related disability, CSI scores, and allodynia, including the relationship between CSI-defined central sensitization and QST-defined allodynia.

## Methods

We conducted a single-center case–control study from June 2023 to December 2025, with approval obtained from the Ethics Committee of Dokkyo Medical University Hospital. Participant recruitment and data collection were completed by September 2025. All participants provided written informed consent for participation in this study. Migraine was diagnosed by headache specialists based on the International Classification of Headache Disorders, 3rd edition (ICHD-3) [11].

Eligible patients were adults aged 18 years or older who were diagnosed with migraine, and were recruited from our outpatient headache clinic. Healthy controls were adults aged 18 years or older without a history of migraine and were recruited from among hospital staff members or family members of patients attending the outpatient clinic. Participants were excluded if they had cognitive impairment or neurodegenerative disease; if preventive migraine treatment had been changed within the previous month, resulting in unstable prophylactic therapy; if a structural brain disease that could cause headache was present; or if medication-overuse headache was present, as defined according to the ICHD-3 [11]. Medication-overuse headache was defined as the use of non-steroidal anti-inflammatory drugs on ≥ 15 days per month or triptans on ≥ 10 days per month for at least 3 months. This study included 130 migraine patients (age, 43.8 ± 13.6 years; 101 F) from our outpatient headache clinic and 130 sex-matched healthy controls (age, 42.4 ± 11.8 years; 102 F) after excluding those with incomplete data (patient group, *n* = 5; control group, *n* = 8).

Clinical information such as age, migraine duration, the presence of aura, and the use of acute and preventive treatments was obtained from the medical records and patients’ headache diaries. Migraine-related disability and interictal burden were assessed using the Migraine Disability Assessment (MIDAS; score range, 0–270) [12] and the Migraine Interictal Burden Scale-4 (MIBS-4; score range, 0–12) [13], respectively. Allodynia was evaluated using the 12-item Allodynia Symptom Checklist (ASC-12) [14]. The ASC consists of 12 items assessing the frequency of allodynia symptoms associated with headache attacks, with the total score ranging from 0 to 24 points. In this study, a score ≥ 3 was used to define the presence of ictal allodynia. Symptoms associated with central sensitivity syndromes were assessed using the Japanese version of the CSI, a self-report questionnaire [15]. The CSI consists of 25 items evaluating health-related and central sensitization syndrome-associated somatic symptoms (total score range: 0–100). A CSI score ≥ 40 has been proposed as a cutoff indicating a high burden of symptoms associated with central sensitization syndromes. Accordingly, the same ≥ 40-point cutoff was applied in this study. Daytime sleepiness and sleep quality were assessed using the Epworth Sleepiness Scale (ESS) [16] and the Pittsburgh Sleep Quality Index (PSQI) [17], respectively, and depressive symptoms were assessed using the Beck Depression Inventory-II (BDI-II) [18]. Restless legs syndrome (RLS) was diagnosed based on a questionnaire and clinical interview according to well-established clinical criteria [19]. Quantitative sensory testing for thermal sensation was subsequently performed in the outpatient clinic.

### Quantitative Sensory Testing (QST)

Thermal QST was conducted during headache-free interictal periods using an Intercross-220 (Intercross Corporation, Tokyo, Japan). The stimulation probe consists of three adjacent plates (23 mm × 38 mm each), resulting in a total contact area of 26.22 cm². Three thermal detection thresholds—the heat detection threshold (HDT), cold detection threshold (CDT), and thermal sensory limen (TSL)—were evaluated using the skin-temperature start mode, in which thermal stimulation begins automatically when the probe temperature equilibrates with the skin surface temperature (heat flux = 0 W/m²). In contrast, the heat pain threshold (HPT) and cold pain threshold (CPT) were evaluated using the temperature-start mode, beginning from a fixed baseline temperature of 32 °C. The examination room temperature was maintained at approximately 27 °C throughout the measurements. Room humidity was not formally recorded but remained within normal indoor clinical conditions. Thermal stimuli were applied to the left volar forearm, approximately 5 cm proximal to the wrist, at a ramp rate of ± 1 °C/s (cooling: −1 °C/s; warming: +1 °C/s). The stimulation site and side were kept consistent across all participants to ensure standardization of measurements. Participants were instructed to press a handheld button at the moment they first perceived a change in temperature (HDT, CDT, or TSL) or first felt pain (HPT or CPT). The device automatically recorded the temperature at the time of the response. Each threshold was calculated as the mean of three consecutive measurements. Temperature changes were restricted to the safety range of 0–50 °C to prevent thermal injury (e.g., burns or frostbite). For the TSL, the thermode alternated between warming and cooling, and the TSL was calculated as the mean temperature difference (ΔT) between the point at which a change in temperature was first perceived in each direction. If a participant did not report sensation or pain within this temperature range, the measurement was treated as missing data. In this study, all participants reported sensation or pain within the predefined safety range; therefore, no QST measurements were missing.

Interictal cutaneous allodynia assessed via QST was defined as an abnormal deviation exceeding ± 1 standard deviation (SD) from the mean thermal pain thresholds of healthy controls. Specifically, participants were classified as having allodynia if they exhibited a CPT value greater than + 1 SD and/or an HPT value less than − 1 SD compared with control values, as described in a previous study [20].

### Statistical analysis

Sample size calculations were performed using G*Power software. A case–control design with a 1:1 ratio of migraine patients to control participants was used. In a previous study, migraineurs presented lower heat pain thresholds on the head compared with controls (43.9 ± 3.2 °C vs. 45.1 ± 3.0 °C) [21]. Assuming a true mean difference of 1.2 °C between groups, a total of 111 patients and 111 control subjects would be needed to detect this difference with a power of 0.80 at a two-sided significance level of 0.05.

Normality of continuous variables was assessed using the Shapiro–Wilk test. As all continuous variables showed non-normal distributions, group comparisons were performed using the Mann–Whitney U test. Comparisons of categorical variables were performed using the chi-square test or Fisher’s exact test. Spearman’s rank correlation coefficient was calculated for correlation analyses. A p value < 0.05 in two-sided tests was considered to indicate statistical significance. Statistical analyses were performed using IBM SPSS Statistics version 30 (IBM SPSS, Tokyo, Japan). Figures were created using GraphPad Prism for Mac (version 8; GraphPad Software, San Diego, CA, USA).

## Results

Table 1 shows the characteristics of the patients and controls. The ESS, PSQI, BDI-II and CSI scores and the RLS rate were significantly higher in migraine patients than in healthy controls. Ictal allodynia (ASC-12 score ≥ 3) was observed in 56 patients (43.1%). Central sensitization assessed by the CSI was observed in 40 migraine patients (30.8%) and 1 healthy control (0.8%) (*p* < 0.001). A total of 49.2% of patients received preventive medication, and 40.8% received CGRP monoclonal antibodies (Supplementary Table 1).

**Table 1:** Characteristics of migraine patients and healthy controls

	Migraine patients	Controls	*p* value
*n* (M/F)	130 (29/101)	130 (28/102)	0.881
Age, years	43.8 ± 13.6	42.4 ± 11.8	0.208
Chronic migraine, n (%)	9 (6.9)	-	
Aura, n (%)	39 (15.0)	-	
Disease duration, years	25.7 ± 14.2	-	-
Prophylactics, n (%)	64 (49.2)		
CGRP mAb, n (%)	53 (40.8%)	-	
MIDAS	17.4 ± 30.2	-	-
MIBS-4	3.5 ± 3.5	-	-
ASC-12	3.3 ± 3.8	-	-
ASC-12 ≥ 3, n (%)	56 (43.1)	-	
ESS	9.2 ± 4.5	7.8 ± 4.3	0.009
PSQI	6.8 ± 3.2	4.4 ± 2.2	< 0.001
RLS, n (%)	26 (20.0)	4 (3.1)	< 0.001
BDI-II	14.1 ± 10.3	7.6 ± 6.9	< 0.001
CSI	35.7 ± 21.7	14.1 ± 8.8	< 0.001
CSI ≥ 40, n (%)	40 (30.8)	1 (0.8)	< 0.001

*CGRP* mAb calcitonin gene-related peptide monoclonal antibody, *MIDAS* Migraine Disability Assessment, *MIBS*-4 Migraine Interictal Burden Scale,  *ASC*-12 12-item Allodynia Symptom Checklist,  *ESS* Epworth Sleepiness Scale, *PSQI* Pittsburgh Sleep QualityIndex, *RLS* restless legs syndrome, *BDI*-II Beck Depression Inventory-II, *CSI* Central Sensitization Inventory

**Fig. 1:**
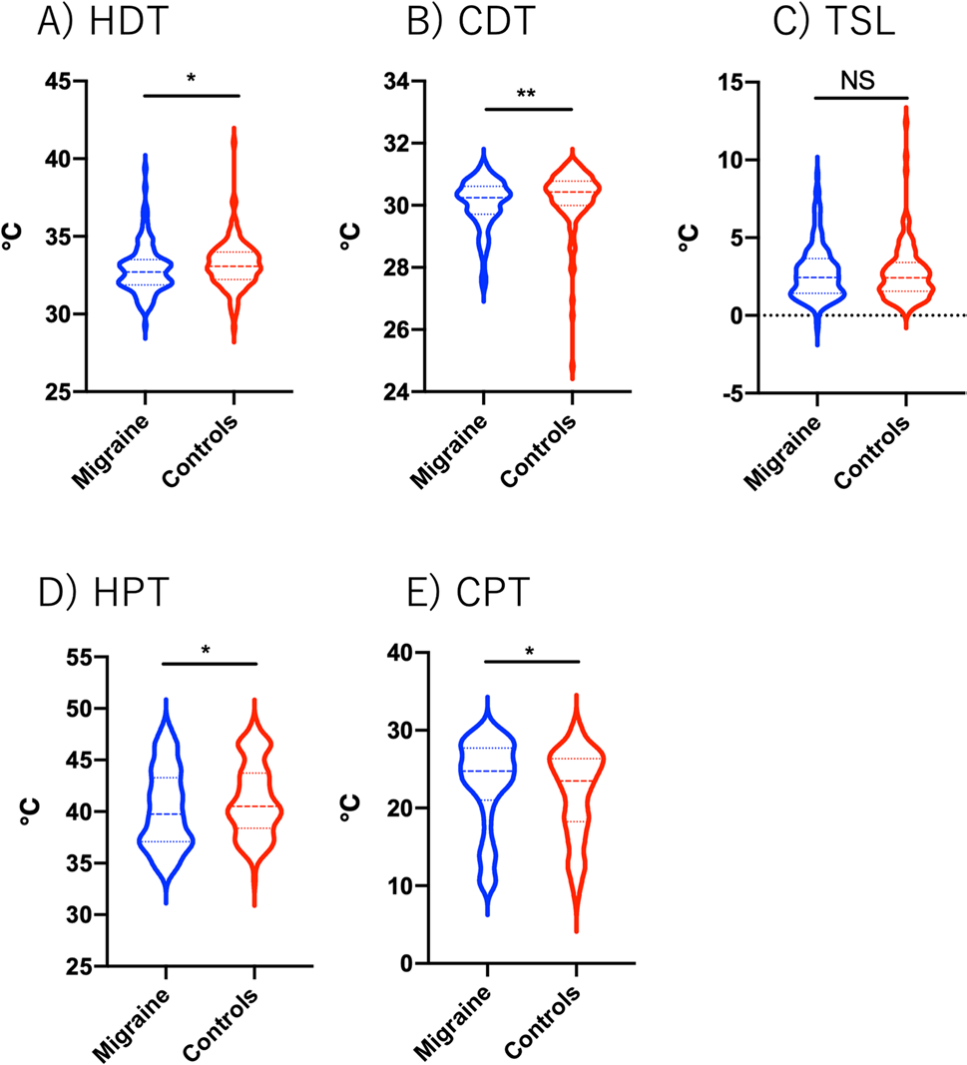
Comparison of quantitative sensory testing parameters between patients with migraine and controls. (**A**) Heat detection threshold (HDT), (**B**) cold detection threshold (CDT), (**C**) thermal sensory limen (TSL), (**D**) heat pain threshold (HPT), and (**E**) cold pain threshold (CPT). Violin plots illustrate the distribution of the data using kernel density estimation. The central line indicates the median, and the dotted lines represent the interquartile range. NS indicates not significant. *p < 0.05 and **p < 0.01

QST differed significantly between patients with migraine and controls. HDT and CDT were significantly different between patients with migraine and controls (Fig. 1A, B). No significant difference in TSL was observed between the two groups (Fig. 1C). The HPT was significantly lower and the CPT was significantly higher in patients with migraine than in controls (Fig. 1D, E). The QST parameters were also compared among migraine patients according to the presence of RLS, poor sleep quality (PSQI > 5), preventive treatment, and treatment with CGRP monoclonal antibodies; however, no significant differences were observed (data not shown).

The CSI score was significantly positively correlated with the ASC-12 score, MIDAS score, MIBS-4 score, PSQI, and BDI-II score (Table 2). With respect to the sensory threshold measures, the CSI score was negatively correlated with the HDT, HPT, and TSL and positively correlated with the CDT and CPT. In contrast, the PSQI, MIBS-4, and BDI-II scores were not significantly correlated with the sensory threshold measures. The MIDAS score was positively correlated with the HDT. The ESS score was negatively correlated with the CDT and positively correlated with the TSL.

**Table 2:** Correlation analysis of the Central Sensitization Inventory score with clinical scales and thermal quantitative sensory testing measures

	CSI	ASC12	MIDAS	MIBS4	PSQI	ESS	BDI-II	HDT	CDT	HPT	CPT
ASC-12	0.566**										
MIDAS	0.270**	0.241**									
MIBS-4	0.352**	0.133	0.398**								
PSQI	0.265**	0.131	0.250**	0.356**							
ESS	0.126	0.117	0.130	0.380**	0.191*						
BDI-II	0.296**	0.148	0.344**	0.473**	0.533**	0.415**					
HDT	-0.104	0.023	0.255**	0.077	0.005	0.164	0.096				
CDT	0.212*	0.199*	0.040	-0.116	-0.041	-0.236**	-0.056	-0.053			
HPT	-0.542**	-0.324**	-0.065	-0.128	-0.046	0.065	-0.086	0.158	-0.225*		
CPT	0.428**	0.282**	0.151	0.128	-0.013	-0.039	-0.028	0.019	0.304**	-0.632**	
TSL	-0.249**	-0.103	0.046	-0.010	0.059	0.233**	0.130	0.297**	-0.625**	0.323**	-0.237**

*CSI* Central Sensitization Inventory, *ASC-12* 12-item Allodynia Symptom Checklist, *MIDAS* Migraine Disability Assessment, *MIBS-4* Migraine Interictal Burden Scale, *PSQI* Pittsburgh Sleep Quality Index, *ESS* Epworth Sleepiness Scale, *BDI-II* Beck Depression Inventory-II, *HDT* Heat detection threshold, *CDT* Cold detection threshold, *HPT* Heat pain threshold, *CPT* Cold pain threshold, *TSL* Thermal sensory limen. The values represent correlation coefficients (Spearman’s rho)

* *p* < 0.05 and ** *p* < 0.01

Interictal allodynia, as detected by QST, was present in 25 migraine patients (19.2%) and 11 healthy controls (8.5%), with a significantly higher prevalence in patients (*p* = 0.012). In migraine patients, ictal allodynia assessed using the ASC-12 (score ≥ 3) predicted QST-defined allodynia with a sensitivity of 72.0% and a specificity of 63.8%. In contrast, a CSI score ≥ 40, indicating a high burden of central sensitization–related symptoms, predicted QST-defined allodynia with a sensitivity of 100% and a specificity of 85.7% in this cohort.

## Discussion

In our study, the lower HPT and higher CPT measured via QST in migraine patients compared with controls indicate hypersensitivity to both heat- and cold-related painful stimuli among the former. Although the difference in tactile hypersensitivity (HDT and CDT) between migraine patients and healthy controls was statistically significant, the magnitude of the difference appeared small, whereas the differences in pain-related hypersensitivity (CPT and HPT) were more pronounced and clinically meaningful, in accordance with the findings of previous studies [20, 22]. These findings support the presence of persistent sensory hypersensitivity in migraine patients beyond the ictal phase. In a study by Sand et al. [23], thermal pain sensitivity in patients with migraine increased during the 24-h preattack period, with a lower HPT and higher CPT than during the interictal phase.

In the present study, thermal pain thresholds in patients with migraine were shifted toward hypersensitivity, whereas compared with those in healthy controls, the sensitivity to cold detection tended to decrease in the migraine group. In addition, although the HDT was lower in migraine patients than in controls, the HDT was positively correlated with disease severity as assessed by the MIDAS score, indicating that patients with a greater migraine burden tended to exhibit reduced sensitivity to innocuous warm stimuli. Similarly, van Welie et al. [24] reported that pain-related measures, such as pressure pain thresholds, were relatively consistently altered in patients with migraine, whereas the CDT and WDT showed less consistent and more variable changes, including elevated thresholds. Together, these observations suggest that sensory processing abnormalities in migraine patients are not uniform across sensory modalities but rather involve differential and nonuniform alterations between nociceptive and sensory detection systems [25, 26]. In addition to alterations in sensory thresholds, enhanced temporal summation of pain, reflecting altered dynamic central nociceptive processing, has been reported in patients with migraine, particularly in patients with a higher attack frequency. Although temporal summation was not assessed in the present study, these findings indicate that sensory abnormalities in patients with migraine extend beyond static threshold measures [25].

In this study, correlation analyses revealed that the CSI score was significantly associated with sensory hypersensitivity assessed by QST, particularly pain-related measures. In addition, the CSI score was significantly correlated with the ASC-12 score, reflecting ictal allodynia, as well as with the MIDAS score and MIBS-4 score, indicating the migraine-related disease burden. Significant associations were also observed with the PSQI and BDI-II scores, reflecting sleep disturbances and depressive symptoms. These findings suggest that the CSI score broadly reflects clinical severity across multiple dimensions in migraine patients. The CSI has been shown to capture a broad cluster of symptoms related to central sensitization, including pain hypersensitivity, sleep disturbances, affective symptoms, and fatigue [8, 27]. In migraine populations, higher CSI scores have been associated with headache-related disability, cutaneous allodynia, and psychiatric comorbidities [27, 28], supporting its relevance as an index of the global disease burden rather than isolated symptoms. In contrast, the PSQI, BDI-II, and MIBS-4 scores were not significantly correlated with the QST parameters. Taken together, these results suggest that the CSI may serve as a useful patient-reported measure reflecting the relationship between migraine-related symptom burden and objective sensory abnormalities assessed using QST.

Furthermore, interictal allodynia detected by QST was more strongly associated with higher CSI scores than with ictal allodynia assessed by the ASC-12. While ASC-12–defined ictal allodynia showed moderate sensitivity and specificity for identifying QST-defined allodynia, a CSI score ≥ 40, indicating a higher burden of central sensitization–related symptoms, showed higher sensitivity and specificity in this cohort. These findings suggest that, compared with attack-based allodynia questionnaires, the CSI may better reflect interictal sensory hypersensitivity in migraine patients.

This study has several limitations. It was a single-center, cross-sectional study, which limits the generalizability of the findings and precludes causal inference. QST was performed only during the interictal period, and phase-dependent changes in sensory processing across the migraine cycle were not assessed. The evaluation was limited to thermal QST parameters, and other dimensions of sensory processing, such as mechanical pain sensitivity or temporal summation, were not examined. In this study, 49.2% of patients with migraine received preventive medications, including CGRP monoclonal antibodies. Although patients with medication overuse-induced headache were excluded, the use of preventive treatments may have influenced QST measures to some extent. In addition, analyses were not adjusted for the duration or timing of preventive therapy. Sensory hypersensitivity has been reported in patients with RLS based on sensory testing [29]; however, in the present analysis, no significant differences in the QST parameters were observed between migraine patients with comorbid RLS and those without RLS. Further studies with larger sample sizes are warranted to clarify these issues. Finally, cranial autonomic symptoms were not evaluated in this study, but previous research has linked such symptoms to cutaneous hypersensitivity and central sensitization [30].

## Conclusions

In this study, QST measurements indicated hypersensitivity to thermal pain in patients with migraine. Furthermore, the CSI may be useful for detecting central sensitization-related sensory hypersensitivity during the interictal period in patients with migraine.

## Supplementary Information

Supplementary Material 1.

## Data Availability

The datasets analyzed in this study are available from the corresponding author upon reasonable request.
