# Supplementary material for: Interictal thermal pain hypersensitivity and central sensitization in migraine patients: a case–control study using quantitative sensory testing
Source: Head Face Med. 2026 Mar 20;22:47. doi: 10.1186/s13005-026-00614-0 (PMC13123026; doi:10.1186/s13005-026-00614-0)
Supplement: Supplementary file 1 — Supplementary Material 1. [file 13005_2026_614_MOESM1_ESM.docx]

**Supplementary Table 1: Preventive treatments used by patients with migraine**

| **Migraine treatment** | **Total (n=130)** |
| --- | --- |
| Preventive treatment, n (%) | 64 (49.2) |
| CGRP mAb | 53 (40.8) |
| Antidepressants | 7 (5.4) |
| Antiepileptic drugs | 3 (2.3) |
| Beta blockers | 2 (1.5) |
| Calcium channel blockers | 6 (4.6) |

**Supplementary Table 2: Contingency tables for the identification of interictal QST-defined allodynia**

**(A) ASC-12 (cutoff ≥3)**

|  | **QST allodynia (+)** | **QST allodynia (−)** |
| --- | --- | --- |
| ASC-12 (+) | 18 (72.0%) | 38 (36.2%) |
| ASC-12 (−) | 7 (28.0%) | 67 (63.8%) |

TP = 18, FP = 38, FN = 7, TN = 67

**(B) CSI (cutoff ≥40)**

|  | **QST allodynia (+)** | **QST allodynia (−)** |
| --- | --- | --- |
| CSI (+) | 25 (100.0%) | 15 (14.3%) |
| CSI (−) | 0 (0.0%) | 90 (85.7%) |

TP = 25, FP = 15, FN = 0, TN = 90

ASC-12 = 12-item Allodynia Symptom Checklist; CSI = Central Sensitization Inventory; QST = quantitative sensory testing; TP = true positive; FP = false positive; FN = false negative; TN = true negative.

Percentages are column percentages based on QST-defined allodynia status.
